# Near-Infrared Hyperspectral Imaging for Non-Destructive Detection of Old Rice in Freshly Milled Rice

**DOI:** 10.3390/foods15152654

**Published:** 2026-07-28

**Authors:** Saranya Workhwa, Rachit Suwapanich, Woranitta Sahachairungrueng, Anthony Keith Thompson, Sontisuk Teerachaichayut

**Affiliations:** 1 Department of Food Technology and Innovation, Faculty of Technology and Engineering, Udon Thani Rajabhat University, Thahan Road, Mueang, Udon Thani 41000, Thailand; saranyaworkhwa@gmail.com; 2 Department of Food Process Engineering, School of Food-Industry, King Mongkut’s Institute of Technology Ladkrabang, Bangkok 10520, Thailand; rachit.ch@kmitl.ac.th; 3 Department of Food Technology, Faculty of Technology, Khon Kaen University, Mittraphap Road, Muang, Khon Kaen 40002, Thailand; wasaha@kku.ac.th; 4 Department of Postharvest Technology, Cranfield University, College Road, Cranfield, Bedford MK43 0AL, UK; keiththompson28@yahoo.com

**Keywords:** rice adulteration, prediction model, qualification, quantification, multivariate analysis

## Abstract

Adulteration of freshly milled rice with rice from older sources is a fraudulent and illegal practice that exploits consumers. The purpose of this study was to develop a rapid and non-destructive technique that can detect this adulteration of milled rice, using near-infrared hyperspectral imaging (NIR-HSI) in the wavelength range of 935–1720 nm. Adulterated samples were prepared by adding old and freshly milled rice at different levels, scanning the mixed samples, and comparing the results with 100% freshly milled rice samples. All samples were divided into calibration and prediction sets for the development of classification and calibration models. Spectral pretreatment methods were tested to develop the optimum models. For qualitative prediction, the best results for differentiation between freshly milled rice and adulterated samples using partial least squares discriminant analysis (PLS-DA) yielded 96.15% accuracy, 92.86% sensitivity, and 100% specificity. For quantitative prediction, the best calibration model for determining the percentage of mixing with old rice using support vector machine regression (SVMR) yielded a coefficient of determination for prediction (R^2^_p_) = 0.90, and root mean square errors of prediction (RMSEP) = 9.10%. These findings demonstrate the potential of NIR-HSI for both qualitative and quantitative analyses in detecting the adulteration of freshly milled rice with old rice. It can be used as a rapid, nondestructive technique for assessing the authenticity of milled rice.

## 1. Introduction

Rice (*Oryza sativa* L.) is one of the most important staple foods, providing the main source of carbohydrates and protein for more than half of the world’s population, especially in Asia and Africa [1]. Global production of rice in 2025 was 542.02 million metric tons. The top-producing countries were India (28%), China (27%), Bangladesh (7%), Indonesia (6%), Vietnam (5%), and Thailand (4%) [2,3]. Rice quality is therefore one of the most important factors affecting consumer health. The standards for rice quality have become stricter over time. This is driven by economic advancements and improvements in the quality of life [4]. Recently, public interest was drawn to the issue of 10-year-old rice in Thailand. The Thai government auctioned large quantities of old rice from its stockpiles [5]. Therefore, there is a real possibility that some parties may try to resell this aged rice to consumers. Consequently, consumers may be at risk of unknowingly purchasing and consuming adulterated rice products. Techniques to detect this are very important now. Adulterated rice may pose a risk to human health and undermine consumer confidence [6].

Rice exhibits changes in its physicochemical properties upon aging, including increased hardness, darker color, reduced aroma, and lower cooking quality due to protein denaturation, starch retrogradation, and lipid oxidation during storage [7,8,9]. In addition to economic concerns, consumption of old rice may be hazardous. Storage under unsuitable environmental conditions, particularly elevated humidity and temperature, can increase the risk of microbial contamination, including aflatoxin-producing molds [10]. Temperature, water activity (a_w_), and moisture content (MC) are the most critical factors affecting aflatoxin production in rice. Rice contamination by aflatoxins is caused by mold growth arising from improper drying and storage conditions [11,12]. Prolonged storage may lead to biochemical changes such as lipid oxidation, resulting in the formation of rancid compounds that negatively affect both sensory attributes and nutritional quality [6,8]. These risks depend on the conditions and duration of rice storage. When old rice is blended with freshly milled rice, these changes are difficult to visually detect [13,14]. Detecting adulteration of freshly milled rice requires traditional techniques such as chemical assays, sensory evaluation, and chromatography; however, these methods are time-consuming, destructive, and impractical for routine or large-scale screening [15]. Therefore, developing rapid, accurate, and non-destructive analytical techniques to classify rice samples and detect adulteration in freshly milled rice is essential for maintaining consumer confidence, quality assurance, trade transparency, and food safety.

Near-infrared spectroscopy (NIRS) coupled with multivariate analysis is used to develop nondestructive and efficient methods to evaluate food, agricultural, and forest products [16]. Also, advances in hyperspectral imaging (HSI) technology have made it a rapid, non-destructive, and high-throughput method for food quality and safety determination [17,18]. HSI can be used to simultaneously acquire spatial and spectral information of food products, offering a viable alternative to traditional, labor-intensive, and destructive assessment methods [19]. NIR HSI significantly enhances food quality and safety. It is also used in areas such as disease detection and quality assessment [16,20,21,22]. Unlike conventional NIR spectroscopy, which analyzes a single point, NIR HSI collects a spectral image from a sample, enabling visualization of spatial variations and chemical compositions at each pixel [23,24,25]. NIR-HSI has been successfully applied to determine food authenticity and detect adulteration in numerous food and agricultural products. These include milk adulterated with chemicals such as boric acid, salicylic acid, glucose, and formalin [26]; whey protein powder adulterated with inexpensive proteins and non-protein nitrogen sources such as maltodextrin, wheat flour and milk powder [27]; whey protein powder contaminated by five adulterants, soy protein powder, corn flour, wheat flour, rice flour, and maltodextrin [28]; wheat flour adulterated with peanuts, walnuts, or benzoylperoxide [29]; tapioca starch adulterated with limestone powder [30]; turmeric powder contaminated with multiple adulterants including corn flour, rice flour, starch, wheat flour, and zedoary [31]; Hashemi rice adulterated with different varieties of Shiroodi, Fajr, and Neda [32]; *Amorphophallus muelleri* blume flour adulterated with suweg [*Amorphophallus paeoniifolius* (Dennst.)] and taro (*Colocasia esculenta*) flour [33]; arabica coffee adulterated with robusta coffee [34]; beef adulterated with lymphatic meat [35] and the discrimination of genuine Thai Jasmine rice from Pathum Thani 1 and Phitsanulok 2 rice varieties [36]. Therefore, the objective of this study was to investigate the feasibility of using NIR-HSI for the detection of old rice in freshly milled rice. To achieve this objective, freshly milled rice samples were mixed with different quantities of old rice samples. Then, freshly milled and combined samples were evaluated using NIR-HSI. Additionally, the integration of hyperspectral imaging and chemometric analysis was investigated to establish both classification and quantification models. A rapid, reliable, and non-destructive NIR-HSI technique could be useful for industrial quality monitoring, authenticity verification, and regulatory control within the rice supply chain.

## 2. Materials and Methods

### 2.1. Sample Preparation

All jasmine milled rice samples used in the current study were purchased from local markets in Thailand and packaged in heat-sealed polyethylene bags. The samples designated as “old rice” consisted of rice grown, harvested, and milled in either 2012 or 2023, and stored at ambient temperature until analysis. The samples designated as “freshly milled rice” were grown, harvested, and milled in 2025. Rice samples from different commercial brands were selected to ensure that the samples represented independent sources and production batches. The proportion of old rice in freshly milled rice ranged from 1 to 99% (*w*/*w*) at equal intervals. Two sets of samples were prepared using the same procedure: each contained old rice added to freshly milled rice. All mixed samples were considered adulterated samples. Additionally, freshly milled rice samples were prepared separately and used in this study. Each mixed sample was placed in a separate zip-lock polyethylene bag and then gently shaken to ensure homogeneity. All samples were stored at 25 °C before measurements.

### 2.2. Sample Properties

Several milled rice properties were evaluated. These are discussed below.

#### 2.2.1. Color

The color of each sample was measured using a colorimeter (Konica Minolta CR 400, Osaka, Japan). Color values were recorded in the CIE L*a*b* system, where L* represents lightness (0 = black, 100 = white), a* represents the red/green axis, and b* represents the yellow/blue axis. Each sample was measured three times, and the results were averaged. The mean values ± standard deviation (SD) of samples in each group were used for comparison.

#### 2.2.2. Moisture Content

The moisture content of each sample was measured using the method described in [37] employing a hot-air oven (BINDER FD 115, Tuttlingen, Germany). Approximately 5 g of milled rice was weighed (*W*_1_), and dried at 105 °C to a constant weight (*W*_2_). The percent moisture of each group was calculated using Equation (1). The data are reported as mean values ± standard deviation (SD) of both groups.

(1) $$Moisture\;(\%)=\frac{\left(W_{1}-W_{2}\right)}{W_{1}}\times 100$$

where *W*_1_ = weight of sample before drying (g); *W*_2_ = weight of sample after drying (g).

#### 2.2.3. Water Activity Determination

Water activity (a_w_) was measured for each sample using a water activity meter (Lab Touch-aw, Novasina AG, Lachen, Switzerland). The average a_w_ of each sample was calculated from three measurements at room temperature (25 ± 1 °C), and the mean values ± standard deviation (SD) of samples from both groups were compared.

#### 2.2.4. Total Starch Content Determination

The total starch content was determined using the method described by [38]. In this analysis, 5 mg of each sample was mixed with 1 mL of 90% dimethyl sulfoxide (DMSO) and heated at 95 °C for 60 min with vortex mixing every 10 min. After cooling, 100 µL of the solution was transferred into a 96-well microplate and mixed with 100 µL of an iodine solution (3.04 g/L in 90% DMSO). The mixture was shaken for 2 min, and 20 µL was diluted with 180 µL of deionized water in a new microplate. Absorbance was measured at 620 nm using a microplate reader. The total starch content was determined using a standard calibration curve and expressed as percent starch (%). The mean values ± standard deviation (SD) of the samples from both groups were compared.

#### 2.2.5. Protein Determination

The total nitrogen content of each sample was determined using Equation (2) in an automatic nitrogen analyzer LECO FP528 (Leco Corp., St. Joseph, MI, USA). The total protein content was calculated by multiplying the nitrogen content of each sample by a conversion factor, 5.95 [10]. All determinations were performed in triplicate, and the results were expressed as mean values ± standard deviation (SD). The mean values of the two groups were compared.

Protein (%) = %Nitrogen × Conversion Factor(2)

#### 2.2.6. Amylose Content Determination

The amylose content of samples was determined following a modified method of [39]. One-hundred milligrams of milled rice was combined with 1 mL of 95% ethanol and 9 mL of 1 M NaOH before incubation for 24 h at ambient temperature. Each sample was then diluted with distilled water to achieve a final volume of 100 mL. One milliliter of 1 M acetic acid and 2 mL of iodine solution were then added to each 5-mL sample. Distilled water was added to obtain a final 100 mL volume. The mixture was agitated and permitted to rest for 20 min before measuring its UV absorbance at 620 nm. The amylose content of each sample was determined using a standard curve of amylose absorbance. The mean values ± standard deviation (SD) of samples from both groups were compared.

### 2.3. NIR-HSI Measurements

**Figure 1:**
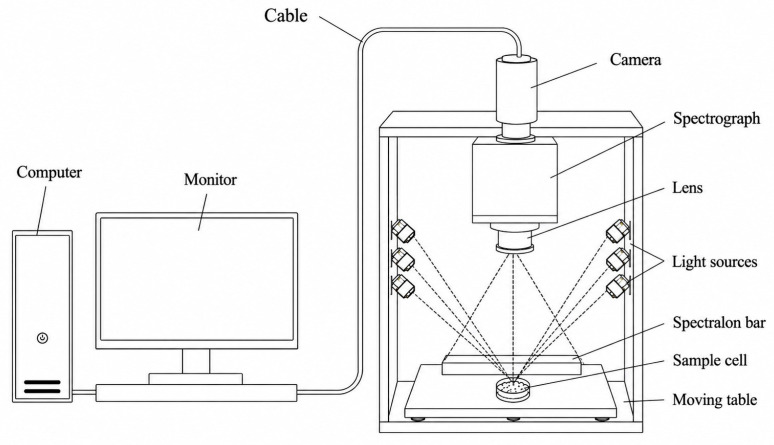
Schematic of the near-infrared hyperspectral imaging unit for evaluating rice samples.

Each sample was filled into a sample cell and then scanned using a near-infrared hyperspectral imaging unit with a Specim Fx17e hyperspectral camera (Spectral Imaging Ltd., Oulu, Finland) over the 935–1720 nm wavelength range. A scan was performed once for each sample. The illumination system consisted of six halogen lamps, with three lamps positioned on each side of the sample at a 45° angle. Each sample was laid on a table moving at 20 mm·s^−1^, as depicted in Figure 1. A dark reference (R_b_) image was acquired while the shutter was closed and the lens was covered. A white reference (R_w_) was obtained using a Spectralon bar before every scan.

### 2.4. Data Analysis

The spectral image of each scan obtained information about the sample and its background. However, only the spectral image of each sample was required for analysis, so the spectral images of the sample cell and the background were removed. A region of interest (ROI) was defined as the spectral image of each sample. Principal component analysis score images were used to identify the ROI by separating the milled rice samples from the background. The resulting score images provided sufficient contrast to distinguish the samples from the background, enabling ROI extraction for subsequent chemometric analysis. Spectral information for each sample was obtained by averaging all pixel spectra within the selected ROI. Therefore, the number of spectra included in the averaging process was equal to the number of pixels in the ROI, and the resulting mean spectrum was used as the representative spectrum of each sample. The calibration and prediction sets were divided at the independently prepared sample level to ensure that spectral information from the same prepared sample was not shared between the two sets. The calibration and prediction sets were also constructed based on the dependent variables. The range of the dependent variables in the calibration set encompassed that of the prediction set. Additionally, the distributions of the dependent variables were comparable between the two sets.

#### 2.4.1. Qualitative Analysis

Principal component analysis (PCA) was employed as an unsupervised multivariate method to reduce the dimensionality of the spectral data while retaining the maximum variance in the original dataset. As reported by [40], PCA transforms correlated variables into a new set of orthogonal principal components (PCs), enabling visualization of data patterns and group separation. It is widely used as a preliminary step before classification. The spectral data of freshly milled and old rice was analyzed using PCA.

Partial least squares (PLS) was employed as a supervised multivariate technique. Partial least squares discriminant analysis (PLS-DA) was used to evaluate sample classification performance [41,42]. Support vector machine (SVM) is a powerful supervised machine learning algorithm that determines an optimal hyperplane with the maximum margin between classes in the feature space. SVM is particularly effective for analyzing high-dimensional, nonlinear, and complex datasets. To address nonlinearly separable data, kernel functions are commonly used to project the input data into a higher-dimensional feature space, where linear class separation can be achieved [43]. Depending on the SVM formulation, model performance can be optimized by adjusting the penalty factor (c) or the Nu parameter (ν), together with the kernel parameter gamma (γ). The SVM models were optimized using a grid-search procedure with 20-fold cross-validation for model establishment. The optimal parameter combination was selected based on the highest accuracy. Outlier detection was performed in this study using principal component analysis (PCA) based on Hotelling’s T^2^ ellipse.

**Figure 2:**
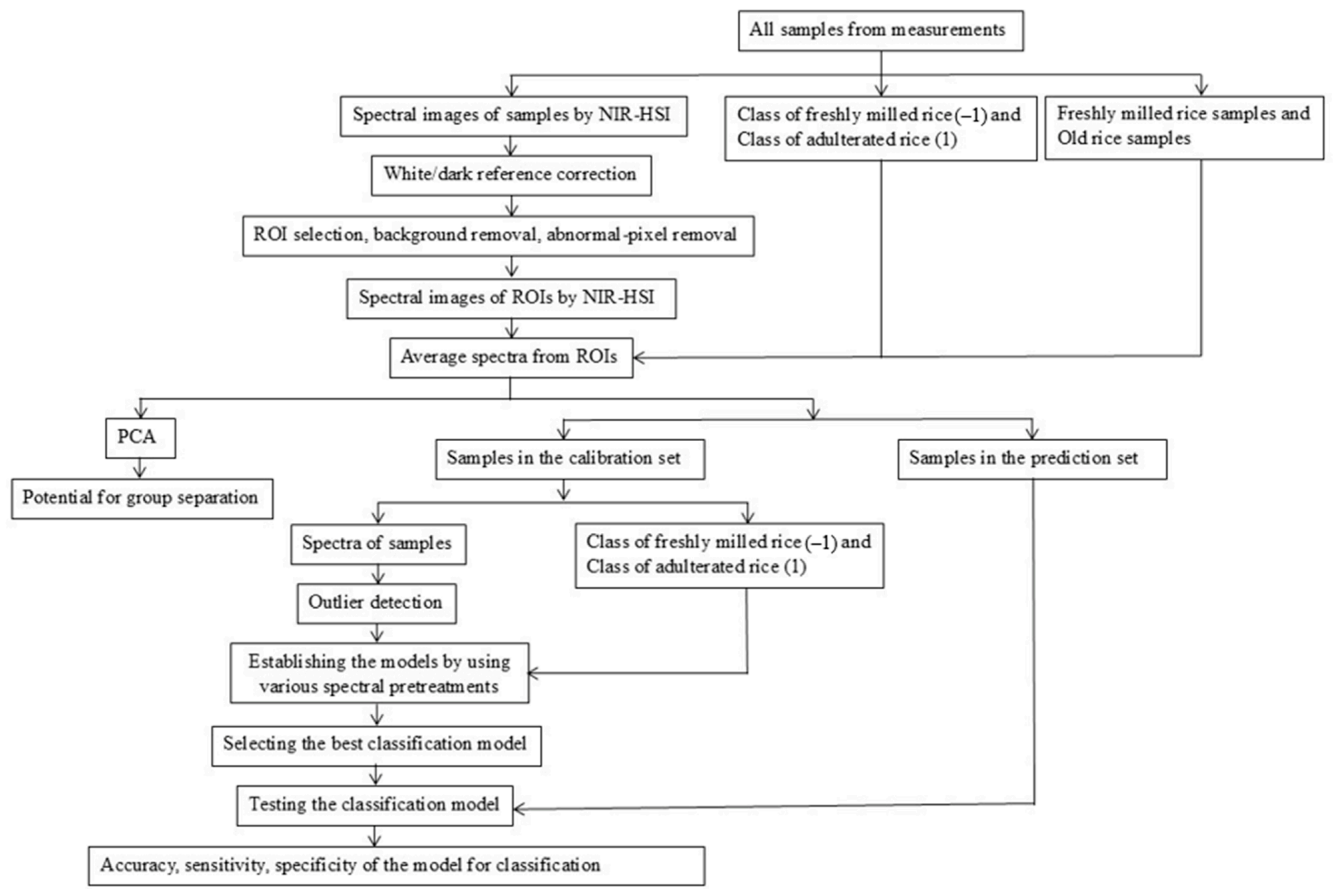
Flow diagram for differentiating freshly milled and adulterated rice.

Support vector machine classification (SVMC) was employed. In this study, PLS-DA and SVMC were used to classify differences between the freshly milled and adulterated rice samples. The procedure for the classification of freshly milled and adulterated rice is shown in Figure 2. Freshly milled and adulterated rice samples were referenced as −1 and 1, respectively. Both samples were used for the calibration and prediction sets. The classification results were determined using a threshold value of 0, and model precision was subsequently evaluated. If predicted values of samples were less than 0 (negative), they were considered freshly milled rice samples. When the values were equal to or greater than 0 (positive), they were classified as adulterated rice samples. Spectral pretreatment methods, including smoothing, 1^st^ derivative, 2^nd^ derivative, standard normal variate (SNV), multiplicative scatter correction (MSC), and their combinations, were evaluated using 20-fold cross-validation in the calibration set to determine the optimal pretreatment method for classification. The classification performance was evaluated using several key metrics, including accuracy, sensitivity, and specificity [44]. These metrics provide a comprehensive evaluation of the classification performance in both the calibration and prediction sets.

In this study, accuracy was defined as the proportion of correctly classified samples (i.e., true positives and true negatives) relative to the total number of samples. An accuracy close to 100% suggested that the classification model was highly effective in correctly classifying the samples according to Equation (3):

(3) $$Accuracy\;(\%)=\frac{\left(TP\;+\;TN\right)}{\left(TP\;+\;TN\;+\;FP\;+\;FN\right)}\times 100$$

Sensitivity (true positive rate) is defined as the proportion of adulterated rice samples that are correctly identified by the classification model and was calculated as

(4) $$Sensitivity\;(\%)=\frac{TP}{\left(TP+FN\right)}\times 100$$

Specificity (true negative rate) was used to measure whether the classification could be used to correctly identify negative samples (freshly milled rice) and was calculated as

(5) $$Specificity\;(\%)=\frac{TN}{\left(TN+FP\right)}\times 100$$

where *TN*, *TP*, *FN*, and *FP* represent the numbers of true negatives, true positives, false negatives, and false positives, respectively.

#### 2.4.2. Quantitative Analysis

**Figure 3:**
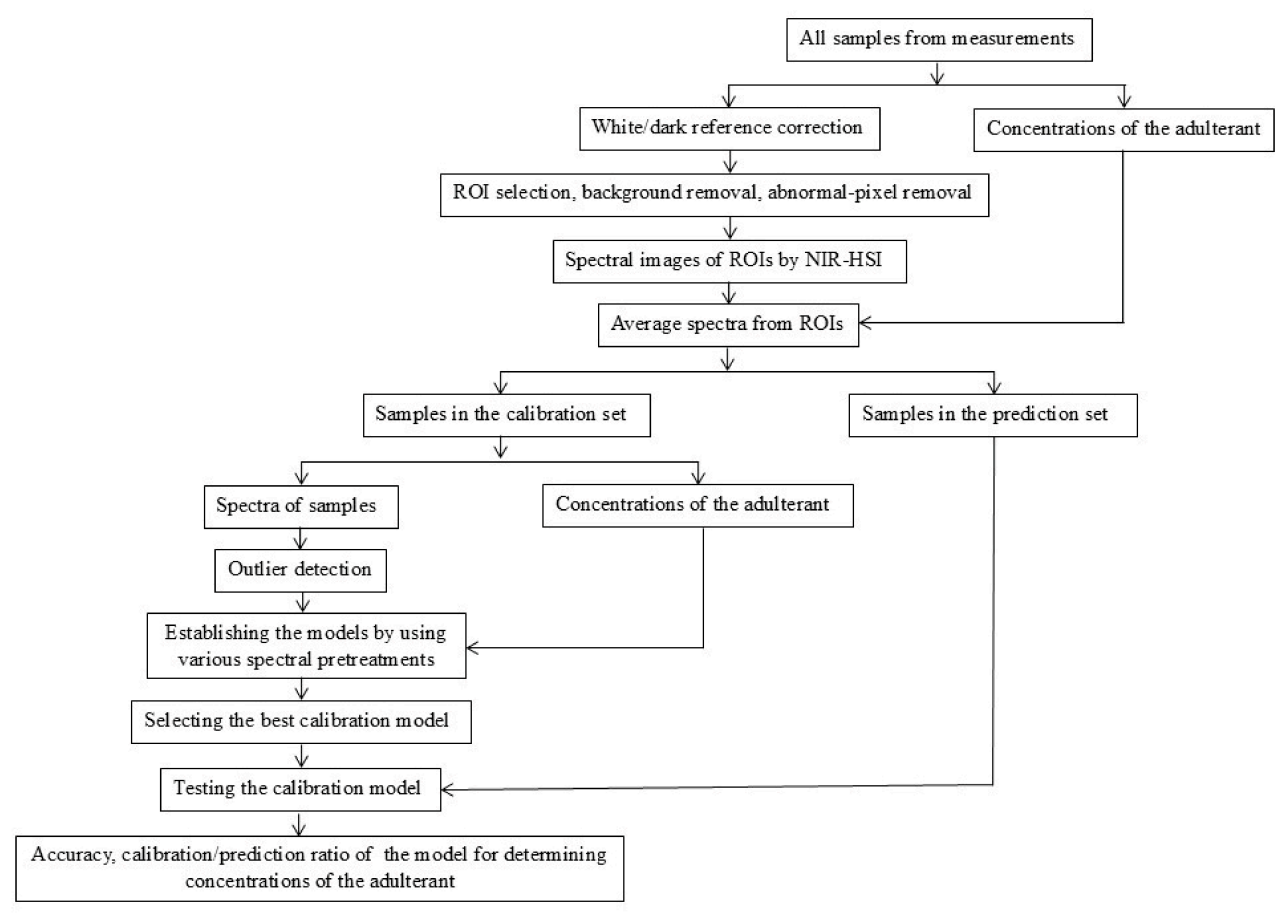
Flow diagram of a calibration model for determining the percentage of old rice in adulterated samples.

The quantification procedure establishing a calibration model for determining the percentage of old rice mixed with freshly milled rice is shown in Figure 3. Partial least squares regression (PLSR) and support vector machine regression (SVMR) were used to develop calibration models to predict the percentage of the adulterant. Freshly milled rice samples mixed with old rice in various ratios were divided into calibration and prediction sets. The spectral pretreatment methods, including smoothing, 1^st^ derivative, 2^nd^ derivative, MSC, SNV, and their combinations, were evaluated using cross-validation within the calibration set. This procedure was performed to identify the optimum spectral pretreatment method for establishing the calibration model. The performance of the calibration models was evaluated using the coefficient of determination (R^2^) and root mean square error (RMSE). High R^2^ values and low RMSE values in both the calibration and prediction sets indicate that the developed model can accurately predict the percentage of rice adulteration.

Statistical analyses were performed using SPSS software (Version 24.0, IBM Corp., Armonk, NY, USA) and The Unscrambler X software (Version 10.5.1, CAMO Software AS, Oslo, Norway).

## 3. Results and Discussion

Color (L*, a*, and b*), protein and moisture content of freshly milled and old rice samples showed significant differences (*p* ≤ 0.05), while water activity, total starch content, and amylose were not significantly different (*p* > 0.05) (Table 1). Although the sample color slightly changed during storage, it was impossible to visually identify.

**Figure 4:**
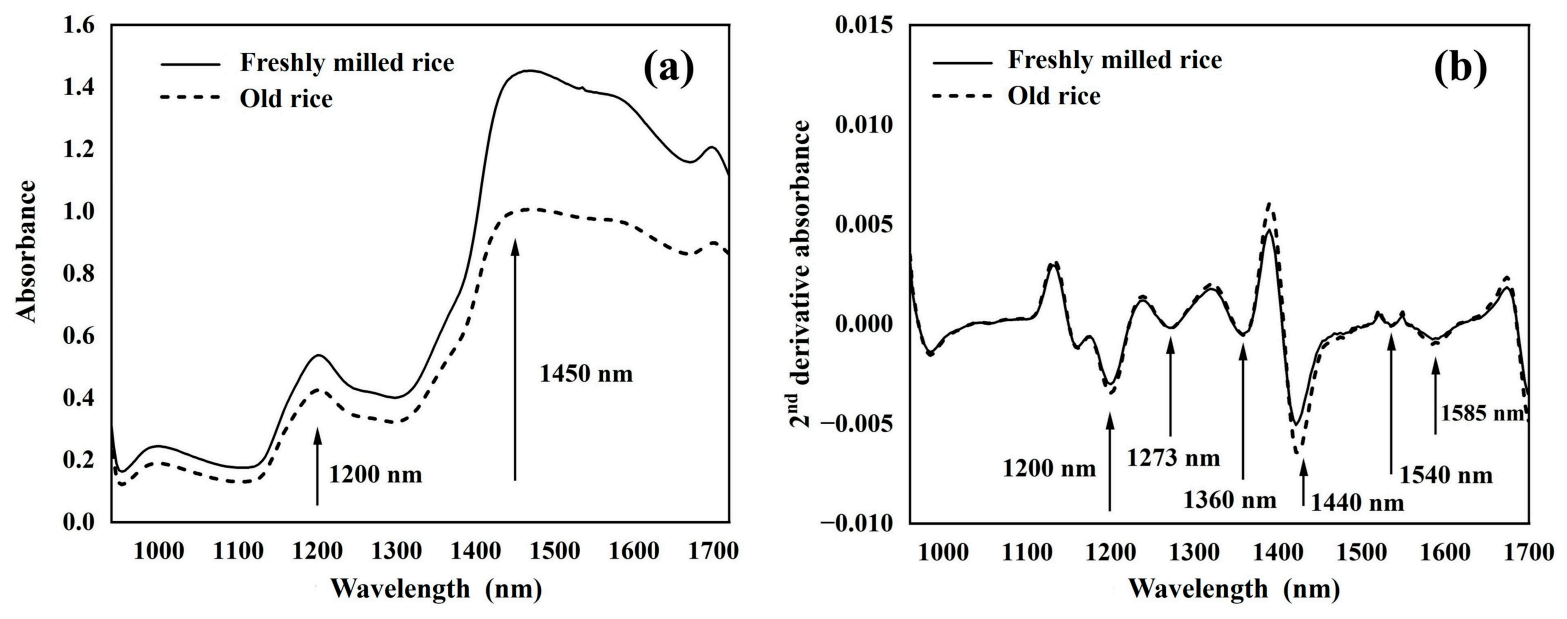
The average absorbance spectra of freshly milled and old rice: (**a**) original absorbance spectra, and (**b**) 2^nd^ derivative absorbance spectra.

The original absorbance spectra of freshly milled and old rice were averaged (Figure 4a), revealing that the large peaks at around 1200 and 1450 nm were influenced by the chemical composition. The absorption band around 1200 nm is attributed to the second overtone of N-H, O-H, and C-H stretching vibrations, while the band near 1450 nm is ascribed to the first overtone of O-H stretching vibrations [45,46]. The 2^nd^ derivative absorbance spectra of freshly milled and old rice were used to identify the main chemical components (Figure 4b), showing peaks at 1200, 1273, 1360, 1440, 1540, and 1585 nm. The peak at 1200 nm reflects the presence of water, corresponding to the second overtone of O-H stretching [45]. The peak at 1273 nm is attributed to the second overtone of C-H stretching vibrations, reflecting the presence of organic constituents containing C-H bonds in both freshly milled and old rice [47].

The peak at 1360 nm arises from C-H combinations, indicating the presence of methyl groups (CH_3_) [48]. The peak at 1440 nm is related to the first overtone of O-H stretching, which is a fundamental structural feature of both water and carbohydrate molecules [47]. The peak at 1540 nm corresponds to the first overtone of O-H stretching, indicating the presence of starch [48]. Additionally, the peak at 1585 nm is attributed to the first overtone of O-H stretching vibrations and is associated with carbohydrates, including starch and glucose [43].

**Figure 5:**
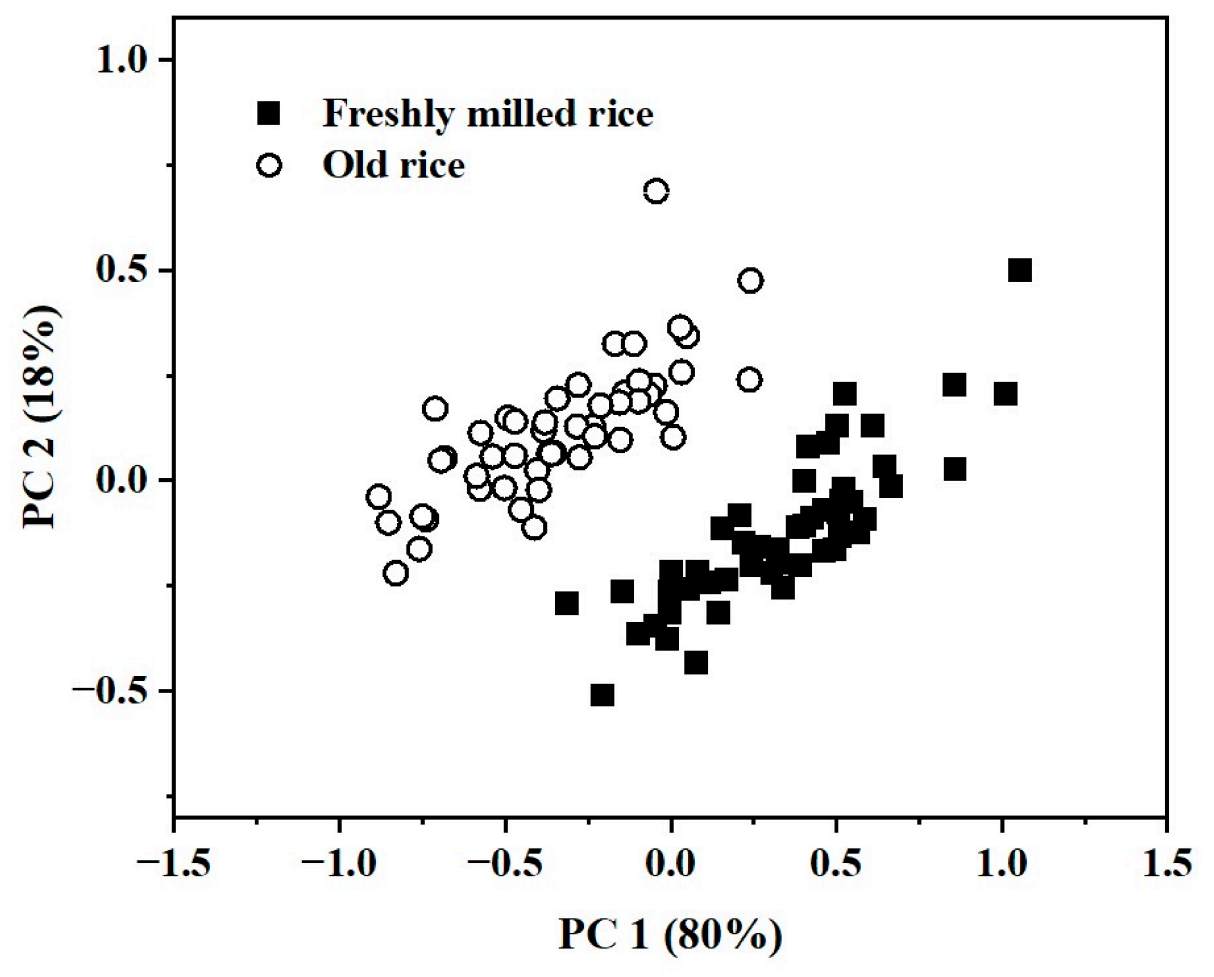
Principal component analysis using the spectral data of freshly milled and old rice.

The score plot of the first principal component (PC1) and second principal component (PC2) explained 80% and 18% of the total variance, respectively. This result showed that group separation using the spectral information of freshly milled and old rice is possible (Figure 5). Substantial differences in color, moisture content, and protein content were observed between freshly milled rice and old rice. However, since color is associated with absorption in the visible wavelength range, it contributed minimally to spectral variation in the NIR region. Instead, water and proteins were the major contributors to NIR spectral variation. Absorption features around 1200 nm were attributed to N-H and O-H bonds, whereas the strong absorption band near 1450 nm was primarily associated with O-H bonds. Consequently, PC1 and PC2 explained 98% of the total variance, indicating that the observed separation between freshly milled rice and old rice was primarily associated with differences in moisture and protein contents. Therefore, spectral data were used for categorical classification with PLS-DA and SVMC in the next step.

### 3.1. Qualitative Analysis for Differentiating Freshly Milled and Adulterated Rice

Freshly milled (N = 198) and adulterated rice samples (N = 234) were combined for analysis using PLS-DA and support vector machine classification (SVMC). All samples (N = 432) were divided into calibration (N = 302) and prediction (N = 130) sets, corresponding to approximately 70% and 30% of the samples, respectively. The standard deviation and the mean value of dependent variables of the freshly milled (−1) and adulterated rice samples (1) in the calibration and prediction sets are similar (Table 2).

**Figure 6:**
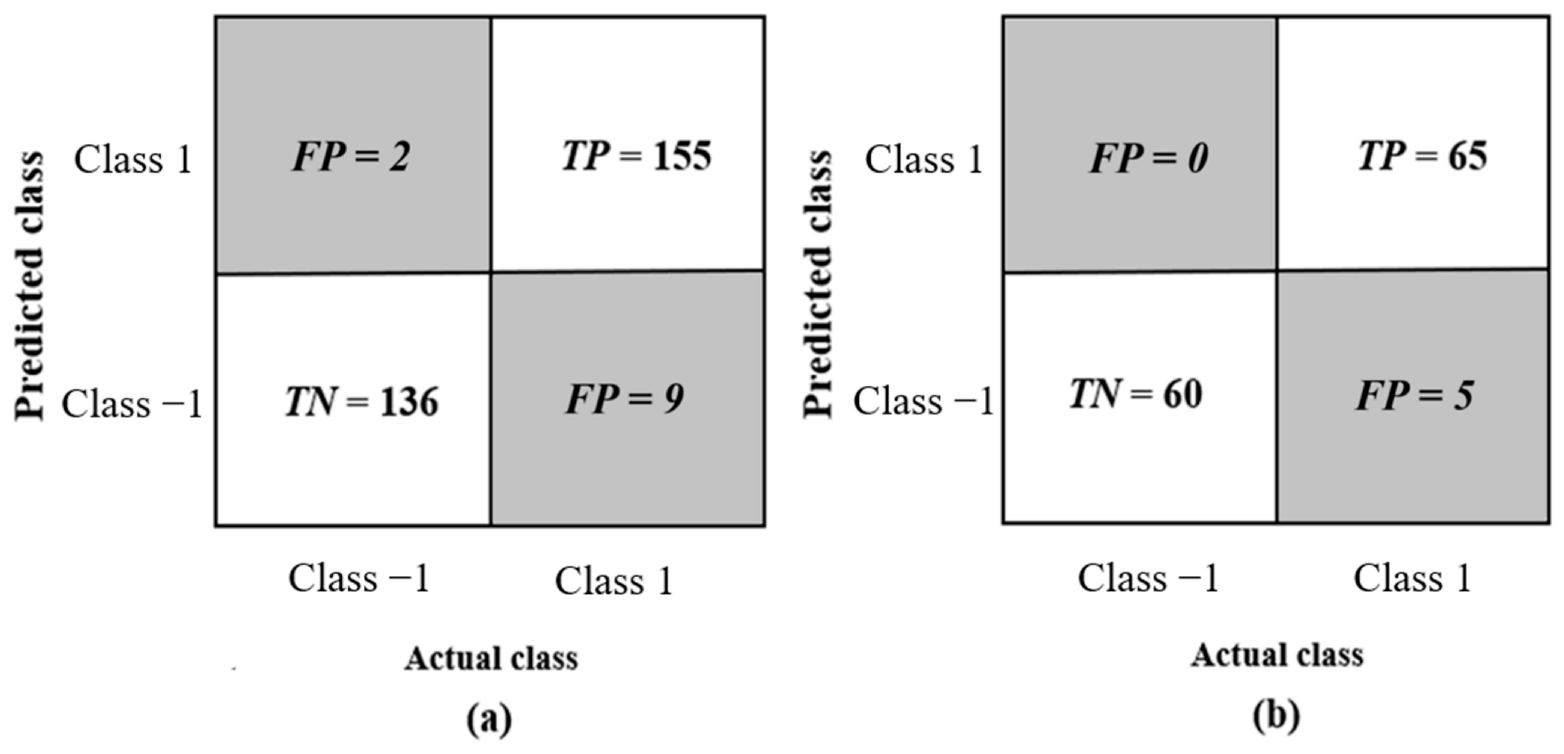
Confusion matrices of classification using PLS-DA: Class −1 is freshly milled rice and Class 1 is adulterated rice in the calibration set (**a**), and the prediction set (**b**).

The original spectral pretreatments provided optimal result accuracy, sensitivity and specificity for PLS-DA (Table 3) as well as optimal accuracy, sensitivity and specificity using the 1^st^ derivative spectral pretreatment for SVMC (Table 4). Original spectral pretreatment could be employed for discriminant analysis using PLS-DA. Also, the 1^st^ derivative spectral pretreatment was selected for discriminant analysis using SVMC. The optimal spectral pretreatments for classification were compared by analyzing samples in the calibration and prediction sets using PLS-DA and SVMC. These results are shown in Table 5. PLS-DA yielded 96.15% accuracy, 92.86% sensitivity, and 100% specificity, while SVMC showed 94.62% accuracy, 95.71% sensitivity and 93.33% specificity. PLS-DA yielded better results than SVMC for classifying the samples into either freshly milled or adulterated rice. A comprehensive evaluation of the classification using PLS-DA was presented by the confusion matrices in both the calibration and prediction sets, as shown in Figure 6.

**Figure 7:**
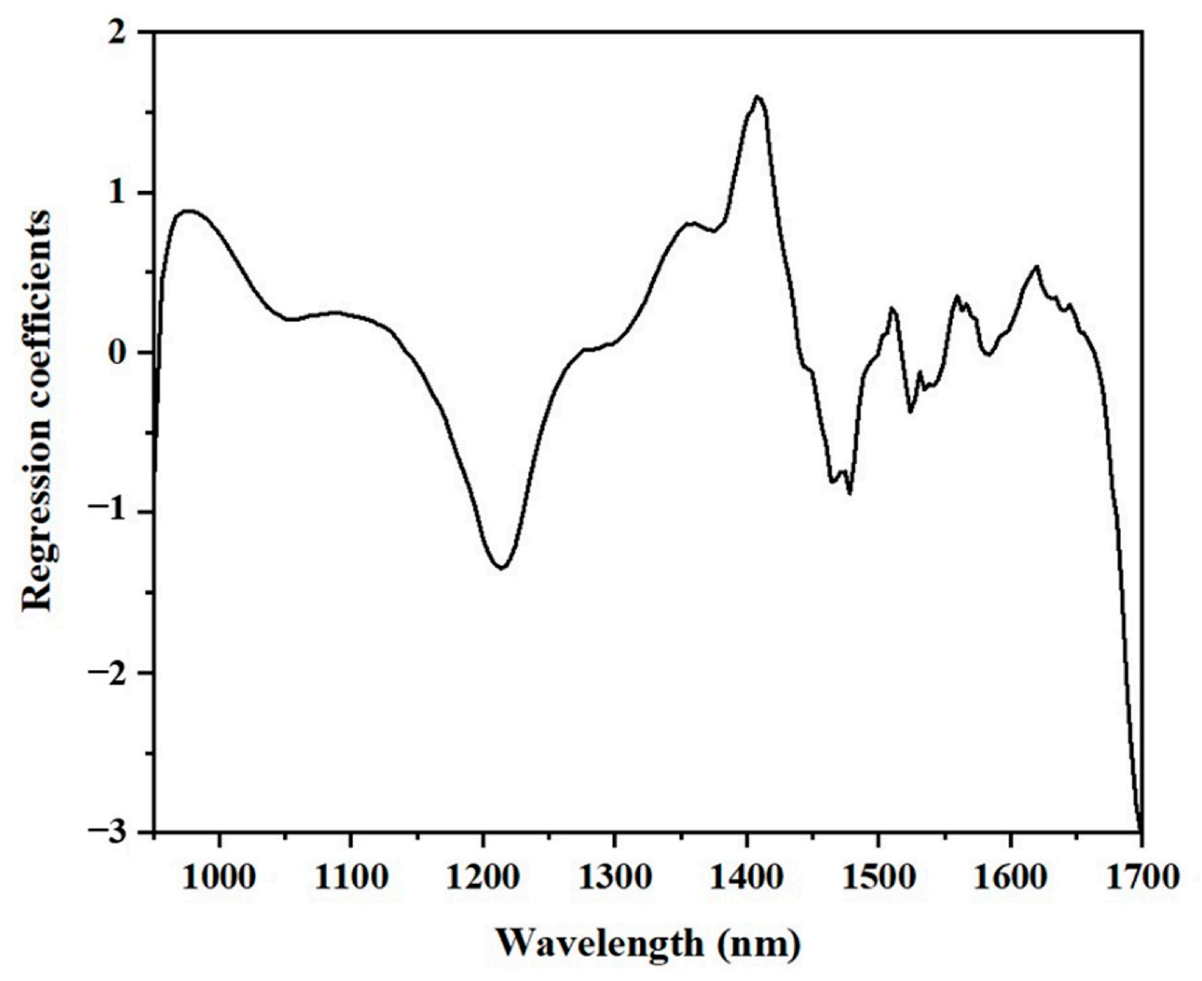
Regression coefficient of the classification model for disgushing freshly milled and adulterated rice.

The wavelength regions around 970–990 nm and 1400–1490 nm are associated with the second and first overtones of O–H stretching vibrations, respectively, indicating water absorption. The spectral region around 1480–1580 nm has been attributed to the first overtone of N–H stretching vibrations, which is related to protein constituents. In addition, the absorption band around 1200 nm is mainly attributed to the second overtone of C–H stretching vibrations, with possible contributions from N–H and O–H vibrations. By examining the regression coefficients of the classification model developed to distinguish between freshly milled rice and adulterated rice (Figure 7), prominent positive peaks were observed around 970, 1400, 1510, and 1550 nm, while negative peaks were observed around 1200, 1480, and 1530 nm, corresponding to the aforementioned absorption bands. These results suggest that O–H and N–H bonds contributed substantially to the model performance, likely reflecting compositional differences between old rice and freshly milled rice, particularly variations in moisture content and protein-related constituents.

### 3.2. Quantitative Analysis for Establishing a Calibration Model for Determining the Percentage of Old Rice Mixed with Freshly Milled Rice

The adulterated rice samples (N = 234), including two freshly milled samples (N = 5) and two old samples (N = 5), were used for quantitative analysis to establish the calibration model. These samples (N = 244) were divided into calibration (N = 171) and prediction (N = 73) sets. Approximately 70% of the samples were assigned to the calibration set, while the remaining 30% were used as the prediction set. The proportion of old rice added to freshly milled rice varied from 0 to 100% in the calibration set. This adequately covered the range of samples in the prediction set (2–99%), and the standard deviations of the two sets were comparable (Table 6). In this study, PLSR and SVMR were used to develop calibration models from 85 samples in the calibration set to predict the mixing percentage with old rice. Various spectral pretreatment methods were investigated to select the optimum calibration models. The optimal calibration models, PLSR and SVMR, were evaluated on 73 samples from the prediction set. The results showed that the optimum root mean square error of cross-validation (RMSECV) by PLSR models was 14.59% using the smoothing spectral pretreatment method (Table 7). Also, the optimal RMSECV by SVMR models was 8.99% using the original spectral pretreatment method (Table 8).

**Figure 8:**
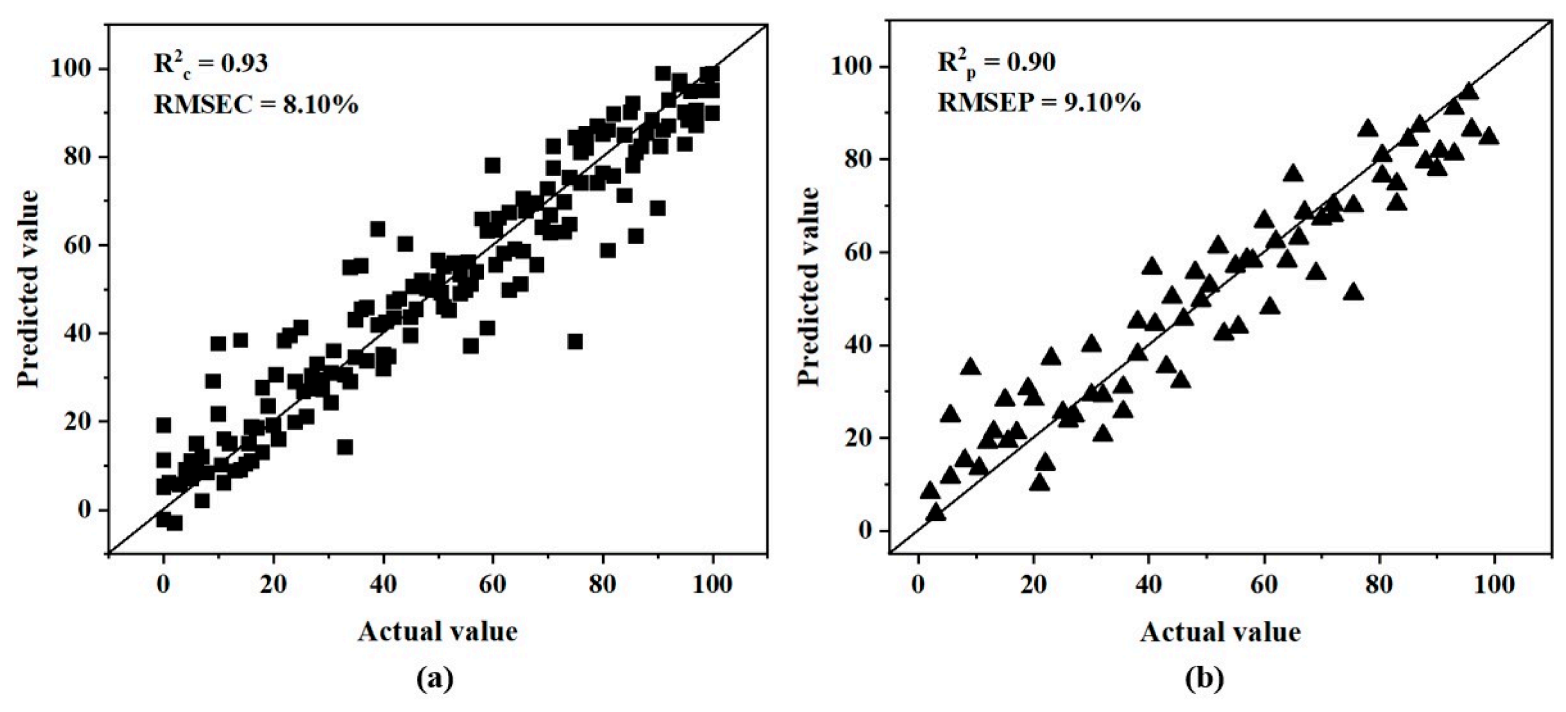
Scatter plots of experimental versus predicted values of the percentage of old rice by the SVMR model in the calibration (**a**), and prediction (**b**) sets.

Based on the above discussion, the smoothing and original spectral pretreatment yielded optimal results for the PLSR and SVMR models, respectively. So, it was selected to establish the calibration models for both. The resulting PLSR and SVMR models were then retested to compare their performance and robustness using samples from both the calibration and prediction sets. These results are shown in Table 9. More accurate results were obtained from the SVMR model. The SVMR model exhibited good predictive performance, with R^2^_c_ and R^2^_p_ values of 0.93 and 0.90, respectively. The R^2^_c_/R^2^_p_ ratio of 1.03 indicated close agreement between the calibration and prediction results, suggesting good model stability and generalization ability. The RMSEC and RMSEP values were 8.10% and 9.10%, respectively, with an RMSEC/RMSEP ratio of 0.89, indicating a low prediction error and a small difference between the calibration and prediction errors. Furthermore, the RPD value of 3.12 demonstrated good predictive capability, confirming that the model was suitable for quantitative analysis. So, it was selected as the best model for predicting the percentage of old rice (Figure 8a,b).

By examining the regression coefficients of the model developed to determine the level of old rice adulteration in freshly milled rice, the highest coefficient was observed around 1200 nm. This suggests that N-H and O-H bonds made important contributions to the model, likely due to differences in moisture and protein contents between old and freshly milled rice samples. These results agree with [49] and show that NIR-HSI can be successfully applied for detecting adulteration in food products, both quantitatively and qualitatively.

## 4. Conclusions

The results demonstrated that near-infrared hyperspectral imaging is a promising technique for detecting the adulteration of freshly milled rice with old milled rice through both qualitative and quantitative analyses under the conditions investigated in this study. From the techniques for prediction testing, the original spectral pretreatment method yielded the best discrimination results with partial least squares discriminant analysis, presenting an accuracy of 96.15%, a sensitivity of 92.86% and a specificity of 100%. The support vector machine regression model with original spectral pretreatment yielded the best results for predicting the percentage of old rice mixed with freshly milled rice, with R^2^_p_ of 0.90 and RMSEP of 9.10%. Since the classification and quantification models achieved good performance, the results demonstrated the feasibility of using near-infrared hyperspectral imaging for industrial monitoring of freshly milled rice authenticity, thereby fulfilling the objective of this study.

## Figures and Tables

**Table 1:** Properties of freshly milled and old rice samples.

Parameter	Color			Moisture Content (%)	Water Activity	Total Starch Content (%)	Protein (%)	Amylose Content (%)
	L*	a*	b*					
Freshly milled rice	74.26 ± 0.45 ^a^	1.04 ± 0.12 ^a^	21.34 ± 0.35 ^a^	11.67 ± 0.89 ^a^	0.36 ± 0.02 ^a^	16.08 ± 0.30 ^a^	7.53 ± 0.12 ^a^	11.60 ± 0.36 ^a^
Old rice	71.35 ± 0.44 ^b^	6.13 ± 0.58 ^b^	27.17 ± 1.07 ^b^	10.26 ± 1.25 ^b^	0.33 ± 0.00 ^b^	15.71 ± 0.39 ^a^	6.59 ± 0.31 ^b^	11.27 ± 0.11 ^a^

Data are expressed as mean ± standard deviation (SD); different letters (a, b) within the same column for each parameter indicate significant differences (*p* ≤ 0.05).

**Table 2:** Characteristics of samples based on dependent variables (−1 and 1) in the calibration and prediction sets for classification analysis using PLS-DA and SVMC.

Set	Number of Samples	Number of Freshly Milled Rice (−1)	Number of Adulterated Rice (1)	Mean (%)	Standard Deviation (%)
Calibration	302	138	164	0.09	1.00
Prediction	130	60	70	0.08	1.00

**Table 3:** Comparison of spectral pretreatments for classifying freshly milled and adulterated rice using a PLS-DA method.

Pretreatment	Factors	PLS-DA ^1^ Method						
		Freshly Milled Rice		Adulterated Rice		Accuracy (%)	Specificity (%)	Sensitivity (%)
		*TN* ^4^	*FP* ^5^	*TP* ^6^	*FN* ^7^			
Original	5	136	2	155	9	96.36	98.55	94.51
Smoothing	5	137	1	151	13	95.36	99.28	92.07
1^st^ derivative	5	135	3	153	11	95.36	97.83	93.29
2^nd^ derivative	3	136	2	150	14	94.70	98.55	91.46
MSC ^2^	5	137	1	149	15	94.70	99.28	90.85
SNV ^3^	5	137	1	148	16	94.37	99.28	90.24
1^st^ derivative + MSC	4	136	2	150	14	94.70	98.55	91.46
1^st^ derivative + SNV	6	135	3	153	11	95.36	97.83	93.29

^1^ PLS-DA = partial least squares discrimination analysis; ^2^ MSC = multiplicative scatter correction; ^3^ SNV = standard normal variate; ^4^ TN = true negative; ^5^ FP = false positive; ^6^ TP = true positive; ^7^ FN = false negative.

**Table 4:** Cross-validation for classifying freshly milled and adulterated rice using an SVMC method.

Pretreatment	Optimization Parameters		SVMC ^1^ Method						
			Freshly Milled Rice		Adulterated Rice		Accuracy (%)	Specificity (%)	Sensitivity (%)
	ν ^4^	γ ^5^	*TN* ^6^	*FP* ^7^	*TP* ^8^	*FN* ^9^			
Original	0.255	0.01	136	2	150	14	94.70	98.55	91.46
Smoothing	0.5	1	134	4	141	23	91.06	97.10	85.98
1^st^ derivative	0.5	0.01	133	5	156	8	95.70	96.38	95.12
2^nd^ derivative	0.5	1	138	0	92	72	76.16	100.00	56.10
MSC ^2^	0.5	1	138	0	143	21	93.05	100.00	87.20
SNV ^3^	0.5	1	137	1	147	17	94.04	99.28	89.63
1^st^ derivative + MSC	0.255	1	69	69	164	0	77.15	50.00	100.00
1^st^ derivative + SNV	0.5	1	138	0	145	19	93.71	100.00	88.41

^1^ SVMC = support vector machine classification; ^2^ MSC = multiplicative scatter correction; ^3^ SNV = standard normal variate; ^4^ ν = Nu parameter; ^5^ γ = kernel function parameter gamma; ^6^ *TN* = true negative; ^7^ *FP* = false positive; ^8^ *TP* = true positive; ^9^ *FN* = false negative.

**Table 5:** Comparison of the classification performance of PLS-DA and SVMC in the calibration and prediction sets.

Method	Pre- Treatment	Factors/ Optimization Parameters	Data Set	Freshly Milled Rice (−1)		Adulterated Rice (1)		Accuracy (%)	Specificity (%)	Sensitivity (%)
				*TN* ^6^	*FP* ^7^	*TP* ^8^	*FN* ^9^			
PLS-DA ^1^	Original	F ^3^ = 5	Cal ^10^ Pred ^11^	136 60	20	155 65	9 5	96.36 96.15	98.55 100	94.51 92.86
SVMC ^2^	1^st^ derivative	ν ^4^ = 0.5 γ ^5^ = 0.01	Cal Pred	133 56	5 4	156 67	8 3	95.70 94.62	96.38 93.33	95.12 95.71

^1^ PLS-DA = partial least squares discrimination analysis; ^2^ SVMC = support vector machine classification; ^3^ F = factor; ^4^ ν = Nu parameter; ^5^ γ = kernel function parameter gamma; ^6^ *TN* = true negative; ^7^ *FP* = false positive; ^8^ *TP* = true positive; ^9^ *FN* = false negative; ^10^ Cal = calibration set; ^11^ Pred = prediction set.

**Table 6:** Characteristics of the dependent variables (the percentage of old rice) in the calibration and prediction sets.

Set	Number of Samples	Minimum Value (%)	Maximum Value (%)	Mean Value (%)	Standard Deviation (%)
Calibration set	171	0	100	50.22	30.25
Prediction set	73	2	99	50.48	28.43

**Table 7:** Cross-validation results for predicting the percentage of old rice using the PLSR model.

Pretreatment	Factors	PLSR Method	
		R^2^_cv_ ^3^	RMSECV ^4^ (%)
Original	7	0.74	15.52
Smoothing	7	0.77	14.59
1^st^ derivative	6	0.72	16.02
2^nd^ derivative	2	0.63	18.36
MSC ^1^	6	0.70	16.60
SNV ^2^	5	0.70	16.61
1^st^ derivative + MSC	4	0.67	17.36
1^st^ derivative + SNV	5	0.69	16.78

^1^ MSC = multiplicative scatter correction; ^2^ SNV = standard normal variate; ^3^ R^2^_cv_ = coefficient of determination of cross-validation; ^4^ RMSECV = root mean square error of cross-validation.

**Table 8:** Cross-validation results for predicting the percentage of old-rice contamination using the SVMR model.

Pretreatment	Optimization Parameters		NIR Hyperspectral Imaging	
	c ^5^	γ ^6^	R^2^_cv_ ^3^	RMSECV ^4^ (%)
Original	100	0.01	0.91	8.99
Smoothing	100	0.01	0.89	10.02
1^st^ derivative	1	0.01	0.82	12.96
2^nd^ derivative	1	0.01	0.83	12.66
MSC ^1^	10	0.01	0.89	10.06
SNV ^2^	10	0.01	0.89	10.06
1^st^ derivative + MSC	1	0.01	0.85	11.79
1^st^ derivative + SNV	1	0.01	0.85	11.77

^1^ MSC = multiplicative scatter correction; ^2^ SNV = standard normal variate; ^3^ R^2^_cv_ = coefficient of determination of cross-validation; ^4^ RMSECV = root mean square error of cross-validation; ^5^ c = penalty factor; ^6^ γ = kernel function parameter gamma.

**Table 9:** Performance of the PLSR and SVMC models in the calibration and prediction sets.

Method	Pretreatment	Factors/ Optimization Parameters	R^2^_c_ ^5^	R^2^_p_ ^7^	RMSEC ^6^ (%)	RMSEP ^8^ (%)	R^2^_c_/R^2^_p_	RMSEC/RMSEP	RPD
PLSR ^1^	Smoothing	F = 7	0.81	0.75	13.20	14.24	1.08	0.93	2.00
SVMR ^2^	Original	c ^3^ = 100 γ ^4^ = 0.01	0.93	0.90	8.10	9.10	1.03	0.89	3.12

^1^ PLSR = partial least squares regression; ^2^ SVMR = support vector machine regression; ^3^ c = penalty factor; ^4^ γ = kernel function parameter gamma; ^5^ R^2^_c_ = coefficient of determination of calibration; ^6^ RMSEC = root mean square error of calibration; ^7^ R^2^_p_ = coefficient of determination of prediction; ^8^ RMSEP = root mean square error of prediction.

## Data Availability

The original contributions presented in this study are included in the article. Further inquiries can be directed to the corresponding author.
